# Multi-Robot Cyber Physical System for Sensing Environmental Variables of Transmission Line

**DOI:** 10.3390/s18093146

**Published:** 2018-09-18

**Authors:** Fei Fan, Gongping Wu, Man Wang, Qi Cao, Song Yang

**Affiliations:** 1 School of Power and Mechanical Engineering, Wuhan University, Wuhan 430072, China; manwang@whu.edu.cn; 2 State Grid Jilin Electric Power Co., Ltd., Baishan Power Company, Baishan 134300, China; qicao.bs@gmail.com (Q.C.); songyang.bs@gmail.com (S.Y.)

**Keywords:** multi-robot, multi-agent, cyber physical system, team/coalition formation, WSN, inspection robot, transmission lines monitoring, smart grid

## Abstract

The normal operation of a power grid largely depends on the effective monitoring and maintenance of transmission lines, which is a process that has many challenges. The traditional method of the manual or remote inspection of transmission lines is time-consuming, laborious, and inefficient. To address this problem, a novel method has been proposed for the Multi-Robot Cyber Physical System (MRCPS) of a power grid based on inspection robots, a wireless sensor network (WSN), and multi-agent theory to achieve a low-cost, efficient, fault-tolerant, and remote monitoring of power grids. For the sake of an effective monitoring system for smart grids, the very research is conducted focusing on designing a methodology that will realize the efficient, fault-tolerant, and financial balance of a multi-robot team for monitoring transmission lines. Multiple testing scenarios are performed, in which various aspects are explored so as to determine the optimal parameters balancing team performance and financial cost. Furthermore, multi-robot team communication and navigation control in smart grid environments are introduced.

## 1. Introduction

The normal supply of electricity is one of the basic requirements of modern society. With the rapid development of the economy, the power grid is expanding rapidly toward the target of a smart power grid. The safe operation and efficient monitoring of transmission lines are facing many challenges [1]. Overhead high-voltage transmission lines are usually distributed in the wild and exposed to bad environments. Such transmission lines suffer from corrosion and lowered safety levels, and may even be damaged [2]. The changing climate will also cause serious damage to the transmission lines, such as conductor galloping and icing, which will jeopardize the supply of electricity [3]. Therefore, it is very important to monitor the transmission lines efficiently.

The normal operation of a power grid largely depends on the effective monitoring and maintenance of transmission lines. The traditional method of manual inspection is time-consuming, laborious, and inefficient [4]. With the rapid development of sensors, robots, and a WSN, more requirements are set for the intelligence of a power grid monitoring system, which bears the advantages described below. Firstly, the intelligent system can run continuously, which is a necessary measure for the fault detection and monitoring of transmission lines; secondly, the system can reduce labor costs; thirdly, the intelligent system can provide high-quality monitoring information.

Currently, some challenges of smart grid monitoring need to be addressed. One of the challenges is to work under harsh conditions, posing greater requirements for sensors, computers, and robots [5,6]. The other challenge is about the continuous operation of the monitoring system without any interruption or failure. These challenges can be dealt with by designing flexible fault-tolerant cyber physical systems with additional components, such as a wireless sensor network (WSN) and multi-robot system.

For monitoring the transmission lines in a remote place, a large-scale sensors network may be required. Therefore, a mobile sensing system may be an appropriate solution [7,8]. In this article, a heterogeneous multi-robot system is applied to a sensing smart grid. The Multi-Robot Cyber Physical System (MRCPS) contains a variety of inspection robots and portable inspection devices to provide a flexible and diversified method of inspection. MRCPS can monitor the conditions of transmission lines, such as power line breakage and galloping, as well as the environmental conditions, such as vegetation occlusion and snow cover. In carrying out task planning and assignments, heterogeneous robots’ abilities were also considered. Based on graph theory, a task-based weighted synergy graph was established to describe the system structure. The robot team is formed based on simulated annealing algorithm. Some incidental challenges, such as the communication and navigation control of a multi-robot system, are also considered. All of the systems are verified by simulation and on-site experiments.

In summary, a heterogeneous robot team is proposed to monitor the state of transmission lines for cyber physical system applications. The main innovations are as follows:

1. Robot technology, WSN technology, and multi-agent theory are integrated to propose a Multi-Robot Cyber Physical System (MRCPS) for the monitoring of overhead transmission lines. MRCPS enhanced the fault tolerance, and reduced the failures and emergencies. By using a smaller number of mobile nodes in comparison with traditional WSN systems, MRCPS achieves a good balance between monitoring performance and deployment costs.
2. A model of a multi-robot task-based weighted synergy graph was established to better understand the interactive relationship between the performance of robots, robots’ social relations, and the capability of the team. In addition, various test scenarios were explored to verify the validity of the system and the preset parameters.
3. A multi-robot team formation strategy is introduced to ensure the optimal structure for a robot team. Additionally, many efforts are made to solve problems regarding the multi-robot team communication and navigation control in a smart grid environment.

The structure of this paper is as follows. Section 2 summarizes the latest research status. Section 3 describes the components of MRCPS and the communication and navigation methods of the robot team in the smart grid environment in detail. Section 4 discusses the developed methods and algorithm. In Section 5, the field experiments for validating the composition and algorithms of the MRCPS are described. The corresponding discussions and conclusions are provided in the last two sections, respectively.

## 2. State of the Art

Since the state of the technology of smart grid monitoring is extensive [9], only the main contributions have been selected and organized into two parts. Firstly, the main detection target parameters and monitoring methods of transmission lines are introduced, because they are in relation to the design of the sensing system. Again, the research progress of the transmission line robot is reviewed, which provides a useful reference for the development of multi-robot team.

### 2.1. Transmission Line Monitoring

The smart grid is a complex cyber physical system with strong electric field and magnetic field and changeable climatic conditions [10,11]. This system is a highly complex nonlinear coupling control system that is greatly affected by external disturbances.

The applications of smart grid monitoring can be found in numerous publications [12,13,14,15,16,17]. The algorithms of such systems involve a control method of traditional proportional integral derivative (PID), fuzzy control, adaptive control, H∞, etc. These control algorithms were proposed based on the environment in which the transmission lines work. The shape parameters, environmental parameters, energy parameters, and spatial parameters of transmission lines were obtained through the monitoring system.

Intelligent, non-contact technology, and remote sensing are the trends of smart grids [18], due to the requirements for reducing accidents and improving the automatic diagnosis of power grids. Therefore, this paper focuses on reviewing the intelligent non-contact detection methods to obtain the parameters of transmission lines. These detection methods can be divided into sensing based on camera, infrared device, and laser radar, by which different parameters can be obtained as shown in Table 1.

#### 2.1.1. Characteristic Parameters of Transmission Lines

(1) Shape parameters

Overhead transmission lines and on-line devices are key to the operation of a transmission grid. The shape parameters of transmission lines and devices are the most intuitive physical parameters [2,7,18]. The aging, corrosion, and breaking of transmission lines and devices can be directly reflected by the shape parameters.

Obtaining the shape parameters is the key target of the inspection of transmission lines. The common shape parameters are as follows.

- The integrity of the line, such as untwisted and broken strand, foreign body coverage, etc.
- Aging, corrosion, breaking, and even loss of on-line devices represented by insulators, clamp, chain suspension, vibration damper, and spacer.
- The lines and devices are sometimes covered by dirt, and they show color variation caused by lightning strike.

(2) Environment parameters

Transmission lines are usually in the wild. The safe operation of a power grid is closely related to environmental changes [19]. The environment parameters of transmission lines include two categories: natural environmental impacts and non-natural impacts.

- The natural environment, including climate change and vegetation growth, poses a great challenge to the transmission grid. Common climatic effects involve rain, snow, strong wind, and lightning strike. In spring, vegetation growth will threaten power supply safety and cause fires.
- Coverage by foreign bodies, such as plastic bags, kites, or civilian unmanned aerial vehicles (UAVs) are the non-natural influencing factors.

(3) Energy parameters

Transmission lines generate strong electromagnetic fields and large heat while transmitting electric energy. Monitoring the electromagnetic field or temperature changes of a transmission grid is beneficial to the prediction and positioning of transmission line faults [20].

(4) Space parameters

The space parameters of transmission lines are mainly related to the spatial changes of transmission lines in a dynamic environment as follows.

- The galloping and sag of the transmission line.
- The displacement and deformation of an on-line device.
- The structural deformation of the power tower.
- Changes in the natural environment such as vegetation growth.

#### 2.1.2. Non-Contact Sensing Methods

(1) Based on visible light imaging

The most important method for the automatic inspection of transmission lines is machine vision monitoring based on visible light [21,22]. The method uses optical sensors to capture the images of transmission lines, on-line devices, and the environment. A variety of parameters of the power grid can be obtained through this method. These parameters include the shape parameters, environment parameters, and space parameters of transmission lines.

(2) Based on infrared imaging

Although most of the monitoring parameters can be obtained by visible light imaging, the energy distribution of the grid cannot be obtained [22]. The detection method based on infrared imaging can effectively overcome this shortcoming. Through the thermodynamic equation, Joule law, and Kirchhoff’s law, the operation data of the power grid can be obtained.

(3) Based on Light Detection and Ranging (LiDAR)

This method can acquire the spatial parameters of transmission lines [23]. In addition, the acquired Light Detection and Ranging (LiDAR) data can be used to achieve the modeling of the transmission network and environmental space [24,25,26].

(4) Other methods

In addition to the above monitoring methods, other methods include:

- The monitoring of energy parameters such as the magnetic field and electric field of transmission lines.
- The monitoring of space parameters based on ultrasonic technology.

### 2.2. Robots for Inspecting Transmission Lines

Most of the existing transmission lines’ monitoring systems are based on wireless sensor network (WSN) technology [27]. In these applications, cameras, infrared imagers, and LiDAR sensors are installed on power towers or other devices in order to measure and detect the objective parameters.

WSNs has the characteristics of being fault-tolerant, flexible, modular, and having low power consumption. On the one hand, the architecture of WSNs are not fixed, so they can be regrouped and expanded. Therefore, the whole network will still be workable, even with structural faults. On the other hand, the embedded technology development, WSNs with low power consumption, have been widely applied, which makes the WSN more clean and environmentally friendly.

However, the network structure and efficiency of a WSN depend heavily on the investment cost. Due to the fixed installation of WSN communication nodes and sensors, a large number of nodes need to be deployed in order to improve the coverage area and system fault tolerance. A traditional WSNs’ monitoring system for a smart grid needs high investment and maintenance costs, and this solution may be prohibitive.

The robot for a transmission line has been developed gradually and has made important progresses and advantages in its application to the smart grid in recent years. Due to the mobility of robots, they can be used as mobile nodes to carry all kinds of sensors to measure dynamically anywhere in a smart grid, which may help reduce the cost of the WSN. They can be used not only for the dynamic monitoring of transmission lines and the environment, but also for power grid maintenance tasks, such as on-line device replacement and cleaning.

In Table 2 of this paper, recent major robots for transmission line monitoring and maintenance are summarized and compared, considering their applications and scenarios. In addition, robots for transmission line maintenance and operation are also used in some applications. However, when regarding power smart grids, the application of a cyber physical system based on a multi-robot team can be considered as a new contribution of this paper.

## 3. System Description

### 3.1. Overhead Transmission Line and Robot Inspection

Overhead transmission lines are exposed to a harsh environment, such as acid rain corrosion and wind-induced vibration, which limit the service lives of lines. Therefore, the importance of the continuous inspection and maintenance of the power grid is definite. For example, China’s power grid has more than 300,000 km transmission lines with a voltage of 220 kV or above, which need regular inspection and maintenance.

**Figure 1:**
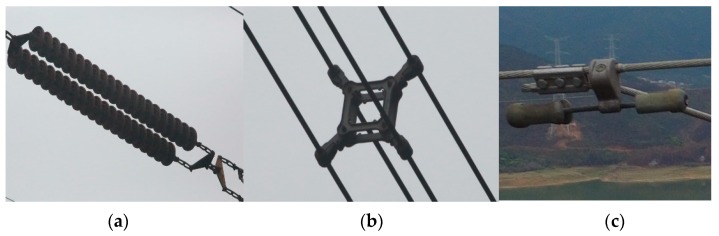
Several common on-line devices. (**a**) Insulators; (**b**) Spacers; (**c**) Vibration damper.

The common on-line devices of transmission lines are shown in Figure 1, including insulators, clamp, chain suspension, vibration damper, spacers, amendments, etc. [2].

Conventional inspection methods for transmission lines mainly rely on manual means for troubleshooting. Inspectors use telescopes, cameras, portable infrared detectors, and small UAVs to inspect power lines in sectional modes. This method requires a lot of manpower, material resources, and time. Generally, it takes weeks or even months to complete the inspection of long-distance transmission lines in remote areas by this method.

**Figure 2:**
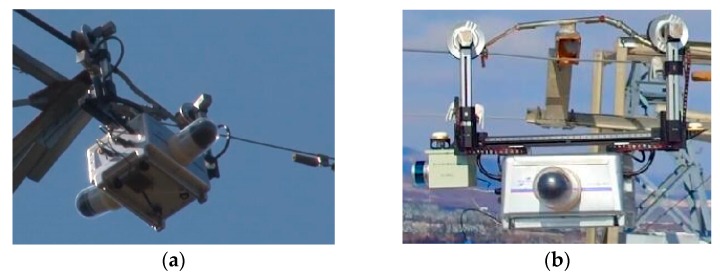
A typical inspection robot for overhead high-voltage transmission lines. (**a**) Obstacle-crossing capacity; (**b**) Automatic inspection by LiDAR method.

As the economy is increasing, transmission lines continue to expand, and in the meantime, the cost of traditional manual inspection is dramatically growing. Therefore, the use of robots has become a considerable alternative, aiming to improve the automation level of power grid monitoring so as to save manpower, cost, and improve the quality of inspection. By using robots, which carry a pan–tilt–zoom (PTZ) camera, infrared detector, laser scanning radar, and other equipment, can be applied in an easier way. A typical inspection robot for the overhead transmission lines that have been developed is shown in Figure 2.

A transmission line inspection robot usually uses two modes (manual and automatic) to conduct transmission lines inspection. The manual method mainly relies on the experience and ability of operators to remote control the robot, so as to realize the remote monitoring of transmission lines. This method is mainly applied to emergency operations. The automatic mode is a method of robot identification and analysis based on existing transmission line information, and automatic operation through specific process. The robot divides the inspection area according to the span between the two power towers, and it completes the automatic inspection of different span sections autonomously.

The steps of the implementing automatic inspection method are as follows.

Step 1: Set up a Task Database (TD) for the inspection mission, including the distance of inspection, the number of segments, the number and type of devices, the power tower type of each segment, etc.

Step 2: When the robot autonomously detects the *K* segment, the *K* segment Detection Sequence (DS) is sequentially obtained.

Step 3: When approaching the target, the robot will decelerate, stop at the checkpoint, and adjust the PTZ camera to capture the image.

- For the target on the same side of the ground line, the external camera is used to capture the image.
- For the target on the opposite side of the ground line, the internal camera is used to capture the image.

Step 4: Store the captured image in the Inspection Database (ID) corresponding to the target parameter.

Step 5: The data of the Inspection Database (ID) and Task Database (TD) are compared and analyzed to assess the operation status of transmission lines.

**Figure 3:**
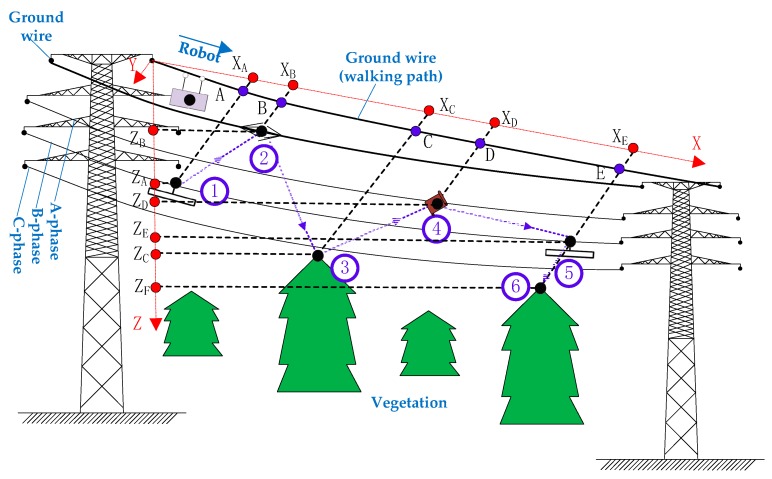
Steps of implementing automatic inspection by inspection robot.

The Detection Sequence (DS) can be generated based on the structure model of the Task Database (TD). As shown in Figure 3, the DS is generated by first using segment numbers to search the TD to get the motion parameters and inspection information on the current segment; then, it assigns a unique number to each target according to their locations. A typical generation of a Detection Sequence is presented in Table 3 for the scene shown in Figure 3.

**Figure 4:**
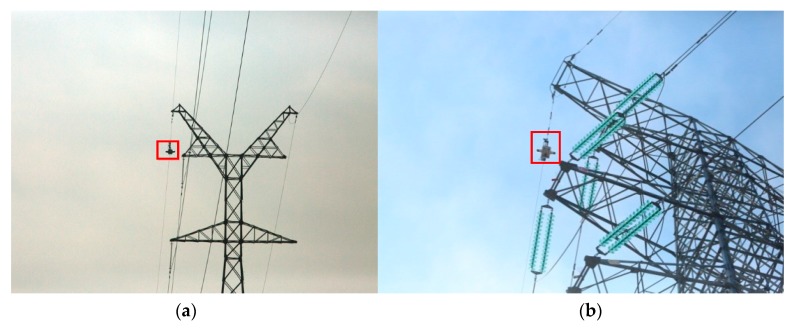
The images of robot crossing different towers. (**a**) Tangent tower; (**b**) Tension support tower.

In order to achieve long-distance inspection and maintenance, robots have to recognize various structures and cross obstacles [8]. Robots usually need to identify and cross two typical obstacles. One is the most common vibration damper and clamp-on lines. The other one is the power tower. Figure 4 shows the images of robots passing through different power towers, from which the difficulty of developing mobile robots across them is demonstrated.

### 3.2. Multi-Robot Cyber Physical System (MRCPS)

As mentioned above, a Multi-Robot Cyber Physical System (MRCPS) for transmission lines is developed and tested. The use of robot teams to replace single robots or traditional WSN systems may provide the following benefits:

1. **Quality:** A robot team works better than a single robot. In the same task, the robots in the team can cooperate with each other for data interaction and integration. Again, based on team detection information, we can make up for the shortage of single test data.
2. **Efficiency:** A robot team is more efficient than a single robot. Under the same conditions, a robot team can call on more resources to complete a task. For example, a multi-robot team can increase coverage space while reducing the operating time.
3. **Flexibility:** A robot team is more flexible than a single robot or WSN sensor system, as it can adapt to different scenarios by changing the organization structure and task assignment of the team.
4. **Cost:** A robot team is more cost-effective than a single robot or traditional WSN monitoring system. On the one hand, the efficiency of a single robot is low, and it needs much time and human assistance (for example, at least two weeks are required to complete the inspection of a 100-km transmission line). A robot team can work autonomously, reducing the working time, and saving the labor cost. On the other hand, a multi-robot system can use a small number of mobile nodes instead of a large-scale WSN system that deploys a large number of sensors.
5. **Fault tolerance:** A robot team can reduce the frequency of failures. If one robot in the team fails, other robots can take over its responsibilities.

In the following sections, we will introduce the robot team members, sensing systems, and communication and navigation control algorithms.

#### 3.2.1. Robots in the Multi-Robot Team

In previous work, different types of robots have been developed and used to inspect and maintain transmission lines [11,12,13,20,24,25,26,27]. The robots are categorized in accordance to their ability of obstacle avoidance. 

**Figure 5:**
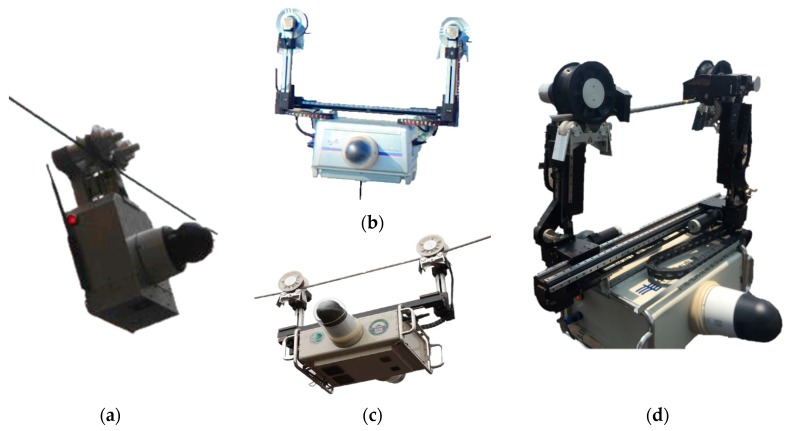
The candidate robots for a multi-robot team. (**a**) Portable Inspection Robot (PIR); (**b**) Traversing Inspection Robot I (TIR I); (**c**) Traversing Inspection Robot II (TIR II); (**d**) Climbing Inspection Robot (CIR).

The first is the Portable Inspection Robot (PIR), which can cross the on-line device, but cannot go through the power tower. The next one is Traversing Inspection Robot (TIR), which has the ability to cross all kinds of on-line devices and some power towers (auxiliary crawling device should be installed on tower structure). The last one is the Climbing Inspection Robot (CIR), which imitates the behavior of animals climbing on the line, and can cross most of obstacles, including on-line devices and power towers. In addition, the integration of small UAVs into the robot team for the inspection of transmission lines was also studied. The proposal of this paper is to apply a team composed of these kinds of robots. The robots are shown in Figure 5, and their main features are described in detail as follows.

**Portable Inspection Robot (PIR).** The PIR is light in weight and easy to transport, but it does not have the capability of trans-power towers. It is commonly used to inspect the operation of transmission lines and devices on a long-distance segment (between two power towers). It is especially convenient for the inspection of transmission lines in rivers and lakes. Compared with other robots, it has the characteristics of miniaturization, light, economy, and energy-saving. It can carry out long-term inspection or emergency inspection for special section lines. PIR with a weight of 15 kg and an average motion speed of 2–3 km/h can carry a 5 kg sensor, such as visible light sensing equipment, infrared sensing equipment, etc.

**Figure 6:**
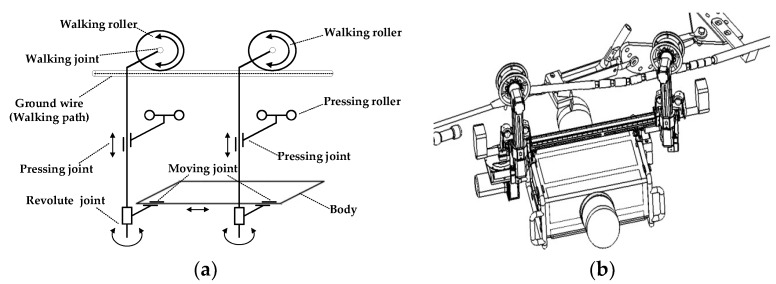
The mechanical structure of the Traversing Inspection Robot (TIR). (**a**) The diagram of the working principle of TIR; (**b**) The obstacle crossing behavior of Traversing Inspection Robot II (TIR II).

**Traversing Inspection Robot (TIR).** The mechanical structure of the TIR is shown in Figure 6. The TIR has two arms suspended on the ground wire, with each arm consisting of a walking joint, a pressing joint, and a rotating joint. The walking joint provides the driving force. The pressing joint allows the robot to adapt to different angles of inclination. The robot can use its arms to move on line. The arms can alternatively release the pressing wheel to cross the obstacles. The TIR has a weight of less than 50 kg, and is able to carry a variety of inspection devices. The average inspection speed is 2.5 km/h. The self-developed TIR has been applied to China’s state grid.

**Figure 7:**
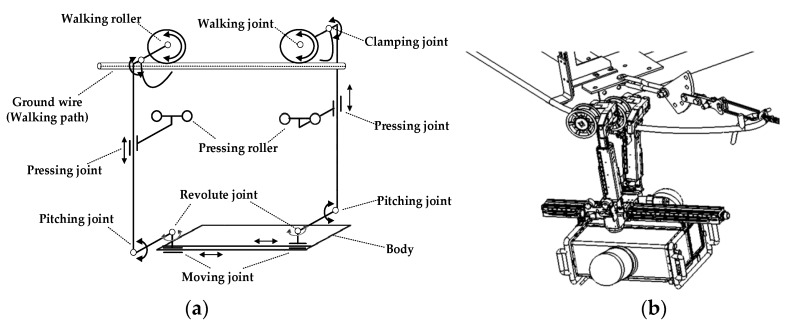
The mechanical structure of the Climbing Inspection Robot (CIR). (**a**) The diagram of the working principle of the CIR; (**b**) The obstacle-crossing behavior of the Climbing Inspection Robot (CIR).

**Climbing Inspection Robot (CIR).** As shown in Figure 7, the CIR has a more complex mechanical structure than the TIR. Compared with the TIR, the CIR has an additional pitching mechanism and a clamping mechanism mounted to the robot’s each arm, and the robot can also perform interleaving behaviors by the two arms. The robot imitates animals’ climbing behavior when crossing obstacles. The following actions are repeated when the robot is crossing an obstacle: single arm grasping line, then crossing arm. The weight is about 60 kg, and it can carry a variety of devices, with an average speed of 1.8 km per hour.

A small UAV is also used as a member of the robot team to increase the flexibility of the overall system. The key parameters of the inspection robots and UAV are shown in Table 4.

#### 3.2.2. Sensors

In the previous chapters, the smart grid sensing methods in different literatures have been discussed. The power grid characteristic parameters and non-contact sensing methods are also summarized.

In this work, a Multi-Robot Cyber Physical System (MRCPS) is designed to measure the variation in the parameters of the transmission line. In different tasks, different sensors can be selected according to the actual situation to obtain the desired data. Especially for small UAVs with limited load capacity and battery capacity, it usually carries only small cameras for short-time inspection tasks.

The robot team chooses the PZT camera (ER580) as the main measuring instrument for the visible light sensing method. In addition, special custom measuring instruments are adopted as well for infrared camera and LiDAR devices. In other measurements, II/DAS-20 measures the tilt angle of the robot so as to calculate the wind direction and wind speed, and the DS18B20 measures the temperature of the robot and environment. All of these sensors can be selectively installed on the Inspection Robot to fit its intended use, especially for infrared camera and LiDAR sensors, which can be installed at both ends of the robot (TIR, CIR) in different applications.

#### 3.2.3. Communication and Navigation Methods

In a previous study [27], we proposed a Robot Delay-Tolerant Sensor Network (RDTSN) for a multi-robot system used in the inspection of transmission grids. Several inspection robots and communication nodes deployed along the transmission line formed an intermittently-connected mobile wireless sensor network. The communication nodes include static nodes (SNs) and wireless central nodes (WCNs). The WCNs are connected to an Optical Fiber Composite Overhead Ground Wire (OPGW).

The main feature of RDTSN is that all of the nodes in the network do not need to maintain real-time communication. Data interaction occurs when a mobile robot approaches other nodes. When the static network is interrupted, the network data is eventually carried by the robot to the nearest WCNs. In addition, the robot will choose a multi-hop transmission path according to the signal strength and transitivity of the nodes in the network.

In addition, a multi-robot navigation and movement method was proposed, namely the Bidirectional Multi-robot Inspection (BMI) [27]. In a multi-robot system, the motion model based on formation control will be more advantageous in terms of reliability and efficiency. Therefore, the BMI model combines the vehicle traffic (two-way traffic rule) and formation control strategy. In the initial state, the members of the robot team are evenly distributed on two ground wires. In the process of motion, each robot will maintain an equidistant formation to maximize the efficiency of inspection. In addition, robots on two ground lines can only move in different directions. This allows the entire robot team to cycle clockwise or counterclockwise along the power line.

The high-voltage ground wire is regarded as the path-constraint *S*, the power tower is considered the constraint point *P*, and the robot is a mobile node.

(1) The coordinates at the unit center are the average of the multiple robots in a short time.
(2) Robots perform uniform distribution in inspection tasks.
  - The whole transmission line is divided into two heterogeneity regions.
  - The robots are evenly distributed on different ground lines in the two regions.
(3) The distance between any two robots on each ground line satisfies *d* ∈ ($\frac{L}{n-1}$ *− a*, $\frac{L}{n-1}$), *n* > 1.
(4) On each path, the moving node can only move in one direction. It moves in the opposite direction on the *S* to *S*’.
(5) At point P, it takes the time of *T_pause_* ∈ (*T_min_*, *T_max_*) to remain stationary or complete an obstacle-climbing process.

In the BMI model, *d* denotes the distance between the two robots on each ground line; *L* denotes the length of each ground line; *n* denotes the number of robots; and *a* denotes the accuracy of formation. All of the robots need to configure their GPS or network-based localization algorithm. The robots can inspect all of the transmission lines around it, including two ground lines. Therefore, the uniform distribution of the robots can achieve the best inspection efficiency.

## 4. Methods and Algorithms

In the previous section, we introduced the inspection process of a single robot in a real-world environment. However, the MRCPS is a multi-robot system. The process of determining the composition and structure of a multi-robot team is directly related to the monitoring capabilities of the MRCPS. In this section, we study the problem of robot team formation, i.e., the generation of an efficient cooperative multi-robot team through robotic capabilities and their social relationships. In addition, in order to make the MRCPS more economical, we tried to find a multi-robot team with a smaller team size and more efficient work.

With references to the literature [28,29,30,31], the method of graph theory to model the multi-robot team is introduced, and the formation of robot team is then deeply studied. In practical tasks, the overall performance of the multi-robot team is not the simple adding of each robot’s working performance. It is often affected by the social relationships between robots, such as communication protocols, software and hardware compatibility, and their performance in tasks. How to form an efficient robot team based on task allocation is a hot topic in the field of multi-agent/robot and cyber physical system [32,33,34], and it is also the focus of this paper.

### 4.1. Robot Performances

**Figure 8:**
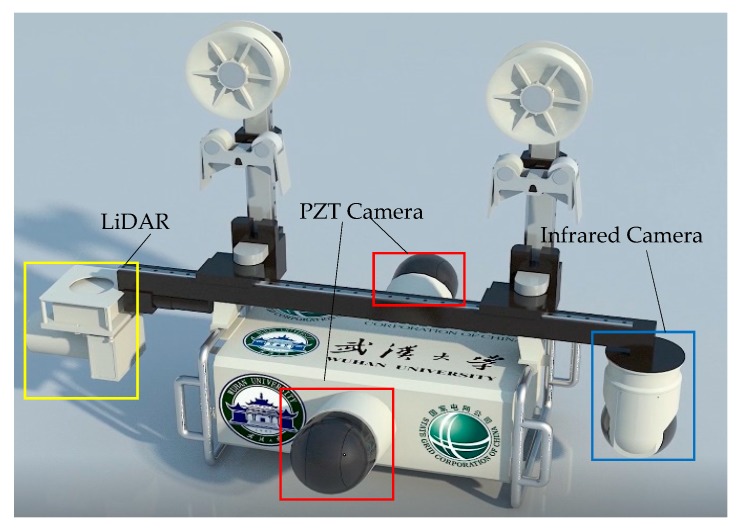
The main sensors and installation of the inspection robot.

Heterogeneous robots have different abilities, which affect their performance in tasks. One way to depict the ability of heterogeneous robots is to assign a value *C_i_* to each robot *R_i_*, where *C_i_* corresponds to the average ability of robot *R_i_* in tasks. In this work, we measure the ability of a robot according to its contribution to overall team performance in a task, rather than in a binary manner (capable/incapable). This method, as shown in Figure 8a, will affect the ability of robots in different missions.

Although the model represents the average ability of all of the robots in tasks, it cannot achieve the comprehensive evaluation under dynamic task scenarios. Since robots are working in a dynamic environment, their performance may vary greatly according to specific scenarios. Since the robot’s ability is in un-modal distribution, its deviation may vary in different tasks. Therefore, we use Normal-Distribution variables *C_i_ ~ N*(*μ*, *σ*^2^) to evaluate the robots’ performance. For example, robot *R_i_* is associated with variable *C_i_ ~ N*(*μ*, *σ*^2^), which is the non-deterministic ability of robot *R_i_* in tasks.

**Figure 9:**
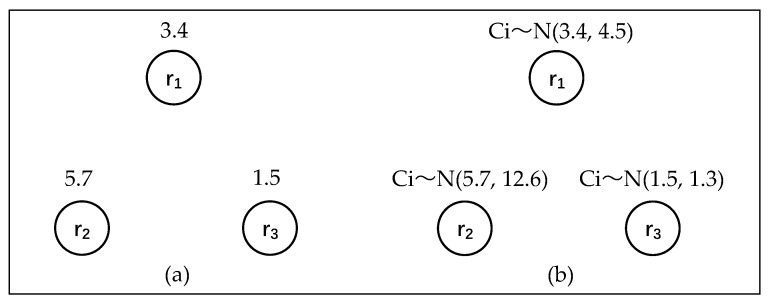
Capability graphs with three agents; (**a**) Values; (**b**) Normally-distributed variables.

Figure 9 gives an example of three heterogeneous robot capabilities, in which Figure 9a reflects the mean capability of the robots in the task. The mean capability of team {*r*_1_, *r*_2_} is better than {*r*_1_, *r*_3_} at this time. Figure 9b uses the normal distribution method to express the distribution of robot capability in dynamic scenes, where r_2_ has higher variance than *r*_3_. Therefore, according to the new evaluation mechanism, {*r*_1_, *r*_3_} may surpass {*r*_1_, *r*_2_}.

### 4.2. Modeling Task-Based Relationships

In order to find out the best scheme of forming all of the robots, it is necessary to establish an accurate social relationship model. In the field of social networking, a social graph is used for the formation of multi-agent teams [29]. The vertices in the graph represent agents, and the vertices are linked by edges to imply the social relationships between them. The weight of edges indicates the strength of the relationship. The focus of this paper is to form a robot team with the best performance in tasks. Therefore, a task-based multi-robot graph model is proposed.

The robot team is represented by the graph *G* = (*V*, *E*), where *V* represents the robot in the team, and E reveals the task-based relationships between those robots (nodes), which is an edge set. The value *e_ij_* represents the communication efficiency between robots, which is used to calculate the weight of the edge (the strength of relationship between robots).

**Figure 10:**
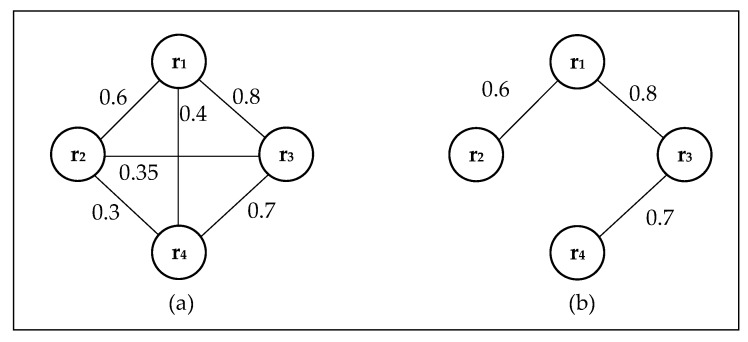
Weighted task-based graphs with three agents. (**a**) A fully connected task-based graph with three agents; (**b**) A simplified task-based graph with three agents.

Figure 10 shows an example of a robot team graph model. In Figure 10a, a fully connected task-based graph is used to represent the strength of task relationships among robots. Each pair of vertices in the graph has edge *E* connections, and different edges give corresponding communication efficiency *e_ij_* (relation strength). A fully connected graph is very complex, i.e., the task-based relationship between the two robots is completely independent, because the relationship weights between robots are arbitrary.

In order to simplify the model used to describe the team relationship, the concept of relationship transitivity is introduced. Figure 10b is a simplified task-based graph model based on the transitivity of social relations. The transitivity of the robots’ communication efficiency is given in Equation (1). The *e_ij_* represents the direct communication efficiency of the robots, and *ie_ij_* represents the indirect communication efficiency. When the weight of the edges in the fully connected graph satisfies Equation (2), the edge *E_ij_* is removed, that is, the weighted task-based graph model is simplified under the premise of keeping the best communication path.

(1) $$ie_{ij}=e_{ik}\prod_{k,j\in E}e_{kj},\;\forall (i,j,k)\in E,i\neq j\neq k,e_{ik},e_{kj}\in \left[0,1\right],$$

(2) $$ie_{ij}\leq e_{ij},\;\forall (i,\;j)\in E,i\neq j,e_{ij}\in \left[0,1\right],$$

(3) $$\phi (r_{i},r_{j})=\frac{d_{\max}(r_{i},r_{j})}{\sum_{i,j\in E}e_{ij}},$$

In the weighted task-based graph, the maximum distance between vertices represents the best communication efficiency as different robots work together. By introducing the Compatibility Function (Equation (3)), which shows the communication efficiency and social relations of robots, we can explicitly model task-based relational models. The Compatibility Function reflects the compatibility between different robots, where *d_max_* (*r_i_*, *r_j_*) is the maximum distance in the graph. There is a positive correlation between the communication efficiency and compatibility of the robot team, so Equation (3) is a monotone increasing function, and a longer distance corresponds to a higher compatibility.

### 4.3. Defining the Weighted Synergy Graph

In the above sections, we have described in detail how the task-based relationship is represented by a compatible function, which uses the distance between the vertices in the graph. Again, we have also introduced how the robot’s ability is represented as a normal distribution variable. In this section, we formally define the weighted synergy graph and introduce how it is used to calculate the performance of the robot team in the task. The weighted synergy graph is defined as follows.

1. *G* = (*V*, *E*) is a connected weighted graph.
2. *V* = *R* = {*r*_1_, *r*_2_, …}, i.e., the set of vertices (*V*) that corresponds to the set of robots (*R*); and *E* = (*r_i_*, *r_j_*, *e_ij_*) is an edge between robots *r_i_*, *r_j_* with an efficiency weight of *e_ij_*.
3. *C_i_ ~ N* (*µ_i_*, *σ_i_^2^*) is robot *r_i_*’s capability at the task.
4. *d_max_* (*r_i_*, *r_j_*) is the maximum weighted distance of a pair of vertices in a relational graph.
5. In the different weighted graphs *G* and *G*’, if *R = R*’, *C = C*’ and the *d_max_* (*r_i_*, *r_j_*) of all vertices are the same, they are equivalent graphs. If Equation (2) is satisfied, the weighted graph of non-maximum *E_ij_* is removed as the simplified weighted synergy graph.

**Figure 11:**
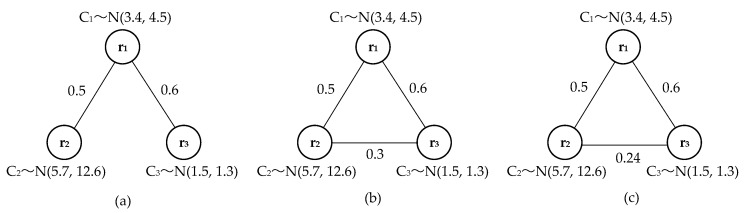
Three equivalent weighted synergy graphs, i.e., the shortest distance between pairs and the capability of robots is equivalent in the three graphs. (**a**) A simplified weighted synergy graph; (**b**) A fully-connected weighted synergy graph; (**c**) The other fully-connected weighted synergy graph.

Figure 11 shows three examples of weighted synergy graphs with three robots, *d_max_* (*r*_1_, *r*_2_) = 0.5, *d_max_* (*r*_2_, *r*_3_) = 0.3, and *d_max_* (*r*_1_, *r*_3_) = 0.6 in all graphs. The three graphs have different structural forms. In Figure 11a, only two edges exist, and the other graphs have three edges. In Figure 11b,c, especially the edge *E*_23_ of Figure 10c is not used, edge *E*_12_ to *E*_23_ can be used to get a better path because it is between *R*_2_ and *R*_3_. Therefore, in general, more than one graphic structure can be used to define the social relationships between robots. When the corresponding robots in the three graphs have the same ability, they are equivalent weighted graphs, where Figure 11a is the simplified weighted synergy graph.

Using the weighted synergy graph, we quantify the performance of the robot team according to the following steps.

Step 1: The robot in team *R* is composed of the minimum synergy team *T* = (*r_i_*, *r_j_*) in every two units.

Step 2: In each minimum synergy team *T* = (*r_i_*, *r_j_*), the synergy ability of two robots is calculated according to the Synergy Function (Equation (4)).

Due to the introduction of social relations, the working ability of each robot in the synergy team is independent, and it is also affected by compatibility. Therefore, the team working ability is the product of multiplying with the Synergy Function after adding the independent robot capability *C_i_* and *C_j_*.

Step 3: The Synergy Function is used in the calculation of the working ability of any synergy team team *R_i_*. The average working efficiency of robots in any team is given by the Efficiency Function (Equation (5)).

Due to the connectivity of weighted synergy graph, the Synergy Function of two robots exists in any team. Therefore, any synergy team *R_i_* of a robot team *R* can be constructed by one or more minimum synergy teams *T*.

(4) $$\psi (r_{i},r_{j})=\frac{d_{\max}(r_{i},r_{j})}{\sum_{i,j\in E}e_{ij}}\cdot (C_{i}+C_{j}),$$

(5) $$\eta (R_{x})=\frac{1}{\left|R_{x}\right|(\left|R_{x}\right|-1)}\sum_{\{r_{i},r_{j}\}\in R}\frac{d_{\max}(r_{i},r_{j})}{\sum_{i,j\in E}e_{ij}}\cdot (C_{i}+C_{j}),$$

(6) $$\mu (R_{x})=\frac{1}{\left|R_{x}\right|(\left|R_{x}\right|-1)}\sum_{\{r_{i},r_{j}\}\in R}\frac{d_{\max}(r_{i},r_{j})}{\sum_{i,j\in E}e_{ij}}\cdot (\mu_{i}+\mu_{j}),$$

(7) $$\sigma^{2}(R_{x})=\frac{1}{{\left|R_{x}\right|}^{2}{\left(\left|R_{x}\right|-1\right)}^{2}}\sum_{\{r_{i},r_{j}\}\in R}\phi^{2}(r_{i},r_{j})\cdot (\sigma_{i}^{2}+\sigma_{j}^{2}),$$

According to the definition of the weighted synergy graph, the average working efficiency of robot team *R* is a Normal-Distribution of random variables *C_ij_ ~ N* (*µ_R_*, *σ_R_*^2^). The mathematical expectation of the working efficiency of the robot team is *µ_R_*, with its variance being *σ_R_*^2^. Their mathematical expressions are shown in Equations (6) and (7).

### 4.4. Optimization and Selection of Multi-Robot Team

#### 4.4.1. Optimization of Robot Team

The weighted synergy graph model introduces the variability of the transitivity of robot relations and the robot’s ability in tasks. In addition, we also define the process of forming the robot team and the method of calculating the average working efficiency of the robot in any team. However, the result of the Efficiency Function is a Normal-Distribution variable. In order to reasonably establish a task-based efficient robot team, these variables need to be sorted, and the following definitions are used.

**Definition** **1:** *Assume that there is an optimized robot team R_ξ_* *⊆* *R, the probability that η(R_ξ_) (team working efficiency) is higher than η_o_ (objective task demand) is ξ, and the probability of any robot team R_x_* *⊆* *R meeting objective task demand η_o_ is no more than ξ. Equation (8) should be satisfied.*

(8) $$P(\eta (R_{\xi })\geq \eta_{a})=\xi \geq P(\eta (R_{x})\geq \eta_{a}),$$

**Inference** **1:** *If there exists the probability ξ of the optimized team R_ξ_* *⊆* *R meeting the objective task demand η_o_ of the task, ξ should satisfy Equation (9).*

(9) $$\xi =1-\Phi (\frac{\mu_{o}-\mu (R_{\xi })}{\sigma (R_{\xi })}),$$

where Φ is the distribution function of the standard normal distribution, and *μ_o_* is the mathematical expectation of objective task demand *η_o_*.

The optimization probability *ξ* of the robot team indicates the probability that the robot optimization team *R_ξ_* exists, and also shows the cost performance and safety of *R_ξ_* in the task. The ξ of the robot team is negatively correlated with the risk of completing tasks; that is, the greater the ξ value, the greater the security of completing the task and the lower the risk. However, a low risk also means a high investment and low return. When the security is too high, the cost performance of the task will degrade. Before the formation of the robot team, the task’s cost performance and safety need to be considered in advance.

Before the optimization of the robot team, the tolerance of the best team to the risk is set in advance according to the actual situation of the task, in order to balance the team’s cost and security. For example, when *ξ* = 0.6 is considered in the task, the best team performs the task in a compromising way. When *ξ* > 0.6, a team with high risk and high reward is more popular. When *ξ* < 0.6, a team with low risk and low economy is more popular.

**Definition** **2:** *The task risk factor of robot optimization team is ρ∈(0, 1), and ρ satisfies Equation (10)*.

(10) ρ=1−ξ, ρ,ξ∈(0,1)

#### 4.4.2. Formation Algorithm of Multi-Robot Team

The main target of our research is to find the best robot team under the given conditions: the task target risk factor (ρ*) and the mathematical expectation of objective task demand (*μ_o_*). Thus, we need to find that the team with better ability is composed of the minimum synergy teams *T* in the Weighted Synergy Graph. The following theorem is presented to analyze the computational complexity.

**Theorem** **1.** *The problem of finding the best team of the MRCPS is non-deterministic polynomial-time hard (NP-hard)*.

**Proof of Theorem** **1.** By definition, a multi-robot team consists of one or more minimum synergy teams *T*. From the unweighted synergy graph, we need to figure out the maximum vertices’ values of the synergy teams. Thus, we turn the problem into a maximum clique problem (MCP) in graph theory to solve it.

Suppose *G* = (*V*, *E*), where *E* = (*v_i_*, *v_j_*), is an unweighted graph. The objective is to determine whether there is a clique of at least n sizes, i.e., a subgraph of n vertices that are completely connected. Through defining a weighted graph *G*’ = (*V*, *E*’), where *E* = (*v_i_, v_j_*, *e_ij_*), *G* can be regarded as a special case of *G’* having equal weights, such as *E* = (*v_i_*, *v_j_*, 1).

Hence, a clique in G corresponds to a clique in G’ that is fully connected with the edges of weight 1. Again, a clique has the greatest synergy (compared with other subsets of size n), because they have the largest number of edges and a weight of 1. Since the MCP is NP-complete, then that of the MRCPS is NP-hard. □

In the process of finding the best team, the optimization probability *ξ* of different team combinations can be calculated, and the task risk factor *ρ* of the robot optimization team is converted to Equation (10). Assuming that the task target risk factor is ρ*, the best robot team R_ξ_* ⊆ *R* is the optimization team of the task risk factor *ρ* matching the target risk factor ρ*. In view of the good performance of the simulated annealing algorithm in the maximum clique problem, we introduce it into a team formation algorithm. In order to optimize and sort the task risk factor *ρ* of the multi-robot team, a Multi-Robot Team Formation Algorithm (MTFA) based on the Simulated Annealing (SA) algorithm is proposed in this paper.

The Simulated Annealing (SA) algorithm is designed to determine the global optimal solution of objective function [35,36]. The MTFA based on the SA does not depend on the selection of an initial value solution; rather, it can find the optimal solution of a target under any initial condition. If the complexity *λ* of the initial robot set *R* is large enough, then the global optimal solution can be determined.

The MTFA is controlled by two layers of loop. The external loop is an iterative process. Given that the complexity *λ*_0_ of an initial robot set and the attenuation factor *α* is less than 1, a variable *k* is used to represent the number of loops of the external loop, and *λ_k_* represents the complexity of the robot team in the *k*th loop. After each external loop, the system is iterated according to the attenuation factor *α*, so the risk factor *λ_k_* satisfies Equation (11).

(11) $$\{\begin{array}{c} \lambda_{k}>0\\ \underset{k\to \infty }{\lim}\lambda_{k}=0\\ \lambda_{k}=\alpha \lambda_{0} \end{array},$$

In the internal loop, the MTFA follows the SA principle and ranks the objective function values. The smaller objective function value is retained in the iteration, the judgment condition is added, and the solution that may be deteriorated is subjected to probabilistic reception. The Metropolis acceptance guideline should be followed:

(12) P={exp{f(Rk)−f(Rk−1)λk},f(Rk)−f(Rk−1)>01,f(Rk)−f(Rk−1)≤0,

where *f* (*x*) is the objective function, *R_k_* is the new solution, and *R_k_*_−1_ is the old solution. The Objective Function of the solution is expressed by Equation (13), and it is the difference between the task risk factor *ρ* of the robot team, and the target risk factor ρ* after *k* iterations.

(13) $$f(R_{k})=\left|\rho^{*}-\Phi (\frac{\mu_{o}-\mu (R_{k})}{\sigma (R_{k})})\right|,$$

The SA method can effectively sort the robot team risk factor *ρ*. However, the running time of the SA varies from tens of minutes to several hours, depending on the complexity of the robot set. Therefore, an adaptive improvement method (MTFA) is proposed by designing a single perturbation rule in the original SA. Equation (14) is the definition of a single perturbation rule:

(14) $$\left\{ \begin{array}{l} f_{num}=\max\{2,\mathrm{int}(\pi \lambda_{0})\}\\ f_{range}=\max\{1,\mathrm{int}(\varepsilon \lambda_{0}C_{\left|R\right|}^{f_{num}})\} \end{array}\right.,$$

where *f_num_* is the perturbation range of the robot’s number, *f_range_* is the neighborhood range of the MTFA perturbation, *int*() is an approximate rounding function, *C_b_^a^* is the permutation combination of the robots set, and *π* and *ε* are custom adaptive strengths. Equation (14) associates the number of the perturbation number and neighborhood range of perturbation with the complexity of robot set.

When the complexity is high, the perturbation neighborhood range is large, and the number of perturbation points is large, which can facilitate the convergence of the algorithm. With the decrease of complexity, the number of perturbation points of the algorithm is reduced, the perturbation neighborhood interval is reduced, and the detail optimization phase is entered. At this time, the optimization result can be further improved. Algorithm 1 shows the pseudo-code of the Multi-Robot Team Formation Algorithm (MTFA).

**Algorithm 1.** MTFA for finding the optimization team.Robot team formation algorithm (*R*, ρ*, *η_o_*) 1: **if** |*R*| > 2 **then**2:   *R_k_* ← Random Team (R)3:   Calculating the objective function *f* (*R_k_*)4:   *f* (*R_ξ_*) ← *f* (*R_k_*)5:   **repeat ()**6:   *R_k−1_* ← *R_k_*
7:   *f* (*R_k−1_*) ← *f* (*R_k_*)8:   *R_k_* ← Neighbor Team (*R_k−_*_1_)9:   Calculating the objective function *f* (*R_k_*) 10:   **if** accept (*f* (*R_k−1_*), *f* (*R_k_*)) **then**11:    *R_k_*_−1_ ← *R_k_*12:    *f* (*R_k_*_−1_) ← *f* (*R_k_*)13:    *f* (*R_ξ_*) ← *f* (*R_k_*)14:   **end if**15:    **until done ()**16:    **return**
*f* (*R_ξ_*) 17: **end if**

Since the size of the target team (n) is not specified, the algorithm is run iteratively for increasing n and has a total run time of *O*(*N*^3^), where N is the size of a robot set. In particular, if the best team size is specified in advance, the algorithm runs in *O*(*n*^2^). In comparison, a brute force algorithm would take *O*(($\begin{array}{c} N\\ n \end{array}$)). Compared with the traditional algorithm, MTFA based on a simulated annealing algorithm can effectively reduce the running time and is more suitable for solving such NP-hard problems.

## 5. Results

The previous sections describe the components, communication, and navigation methods of the Multi-Robot Cyber Physical System (MRCPS). Section 4 also researches the robot team weighted synergy graph model and proposes a Multi-robot Team Formation Algorithm (MTFA) based on graph theory and the Simulated Annealing (SA) algorithm. In this section, the aforementioned method is employed to capture the robot’s team performance in real-world scenarios. The organization of this chapter is as follows: Section 5.1 establishes a model of the robot team in a real-world scenario. Section 5.2 describes testing of mimic transmission line scenarios and provides an analysis and discussion of the experimental results.

### 5.1. Modeling and Simulation of the MRCPS

The MRCPS should autonomously and periodically cover transmission lines to monitor environmental variables. This paper initiates a task-based multi-robot team model for the maintenance and monitoring of transmission lines. The system composition plan is changeable for different tasks.

The inspection and communication capabilities of a multi-robot team should meet the objective task demand, and their values can be obtained by previous experience. In this paper, the inspection time *t* is used to describe the efficiency of robot inspection. The mathematical expectation of the robot team’s objective task demand in different inspection tasks satisfies Equation (15). The working ability of each independent robot is expected to satisfy Equation (16), and the variance of working ability can be obtained by previous experience.

(15) $$\mu_{o}(t_{o},L,n_{o})=n_{o}\frac{L}{t_{o}\left|R_{x}\right|},$$

(16) $$\mu_{i}=\left\{ \begin{array}{ll} v\tau \frac{t_{w}}{t_{o}}, & t_{w}\leq t_{o}\\ v\tau , & t_{w}>t_{o} \end{array}\right.,$$

(17) $$C_{e}=C_{s}/C_{t},$$

In Equation (15), *n_o_* denotes the number of targets for inspection tasks, *L* denotes the mileage of inspection routes, *t_o_* denotes the required inspection time, and *|R_x_| = N* denotes the number of robots in the team. Equation (15) is a function that takes the duration of inspection *t* as an independent variable. It can alter the objective demand of the task by changing the value of parameters. In Equation (16), *v* denotes the motion rate of a robot, *t_w_* denotes the nu-refueled inspection time, and *τ* denotes the average inspection time length for a single target. The communication efficiency between robots is obtained by Equation (17) where *C_e_* denotes the communication efficiency, *C_s_* denotes the communication control semaphore, and *C_t_* denotes the total amount of communication.

A task-based multi-robot team model aims at different task demands. The optimal team is formed on the basis of the weighted synergy graph and the algorithm of forming a robot team. The team performs real-time or delay tolerant communication with other wireless nodes in the inspection task. The uniform distribution of the motion model is used for the inspection of transmission lines. The specific steps for forming a multi-robot team are as follows.

Step 1: Analyze and determine the objective demand of the robot inspection task of transmission lines.

Step 2: Calculate the robots’ working ability and communication efficiency, and establish a weighted synergy graph of the multi-robot team based on the task.

Step 3: Use the Multi-Robot Team Formation Algorithm (MTFA) to find the best team for an objective task demand.

Step 4: Establish an inspect task database and assign the inspection task of each robot inspection task in the team.

Step 5: Form a wireless dynamic sensor network with the heterogeneous multi-robot team and the static nodes for the transmission line monitoring.

Step 6: The robots adopt a uniformly-distributed motion model BMI, and the transmission line inspection by multi-robot team is conducted.

Equation (18) is used to calculate the effectiveness of forming the multi-robot team.

(18) $$Effectiveness(R_{x})=\frac{\left[\rho^{*}-|\rho^{*}-\rho (R_{x})|\right]-\rho (R_{\min}^{*})}{\rho^{*}-\rho (R_{\min}^{*})},$$

where ρ* is the objective task risk factor, *R_min_* is the robot team with the minimum risk factor obtained under the objective task demand, and *R_x_* is the select robot team.

Then, the robot team formation effectiveness is a value from 0 to 1, where 1 is the target team and 0 is the worst performance team. We use the team formation effectiveness to measure the difference in task performance between the robot team and the target team to further evaluate the practical role of the weighted synergy graph model.

Two experiments are designed to verify the effectiveness of the weighted synergy graph model and Multi-robot Team Formation Algorithm (MTFA). In the first experiment, a comparison is performed on the effect of the weighted synergy graph model and the unweighted synergy graph model on the robot team. The second experiment compares the performance of the MTFA with that of the original SA. The total number of robots in the robot set is *N*. For each *N*, the robot sets are tested 100 times. The MTFA parameters are configured as follows: complexity of initial robot set *λ*_0_ = *N*, attenuation factor *α* = 0.95, adaptive strength *κ* = 1, and adaptive strength *ε* = 0.1.

**Figure 12:**
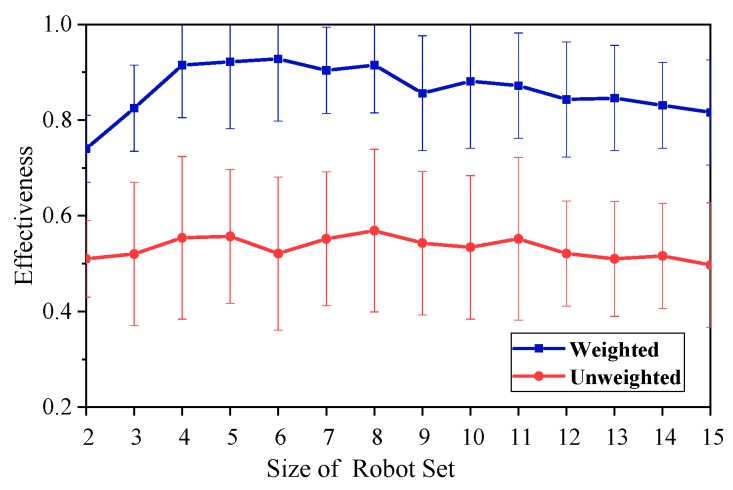
Effectiveness of teams found with the weighted or unweighted synergy graph and Multi-Robot Team Formation Algorithm (MTFA), using simulated annealing with 1000 iterations.

**Figure 13:**
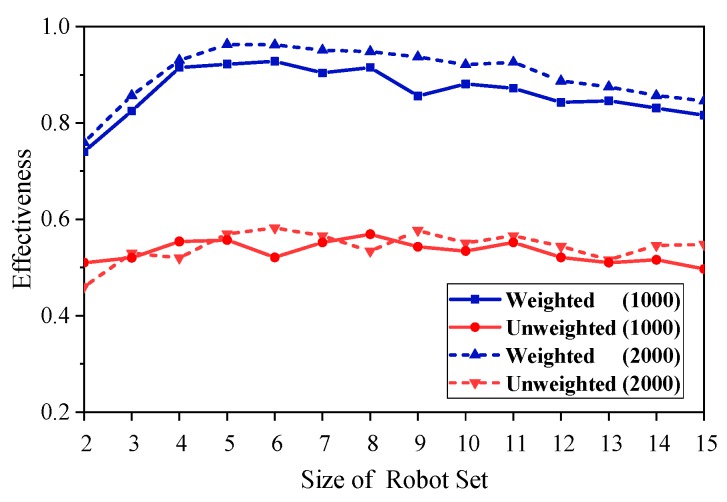
Effectiveness of teams found with the weighted or unweighted synergy graph and MTFA, using simulated annealing with 1000 and 2000 iterations.

Figure 12 and Figure 13 show the effectiveness of the team found in the weighted and unweighted synergy graph, where Figure 12 uses 1000 iterations and Figure 13 uses 2000 iterations.

As can be seen from Figure 12, the performance of the weighted graph model is superior to that of the unweighted graph model. In addition, with the growth of the initial size of the robots’ set, the two models tend to exhibit similar properties, which are described below. As the growth of the robot set increased, the effectiveness of the two methods is firstly improved rapidly (in the range of the size of robots from two to four), and then relatively stabilized with a slight downward trend.

As shown in Figure 13, the performance of a 2000 iteration is better than that of a 1000 iteration, whether using the weighted graph model or the unweighted graph model. Furthermore, under different conditions, the curves of effectiveness show a similar trend, i.e., first increasing rapidly and then declining gently.

While the size of the robots set is small, especially from two to four, the optimal solution and the target have a large error, although the two methods can quickly find the optimal team, and the error of the solution decreases gradually with the set increasing. Therefore, when the size of the robots set is from two to four, the effectiveness of the two models increases rapidly with the increase of the set.

As the size of the set increases further, the difficulty of calculating the optimal solution is gradually increasing, and it is more and more constrained by the computational time and the number of iterations. Therefore, the effectiveness curve shows a slow downward trend, and the group with more iterations obviously perform better.

**Figure 14:**
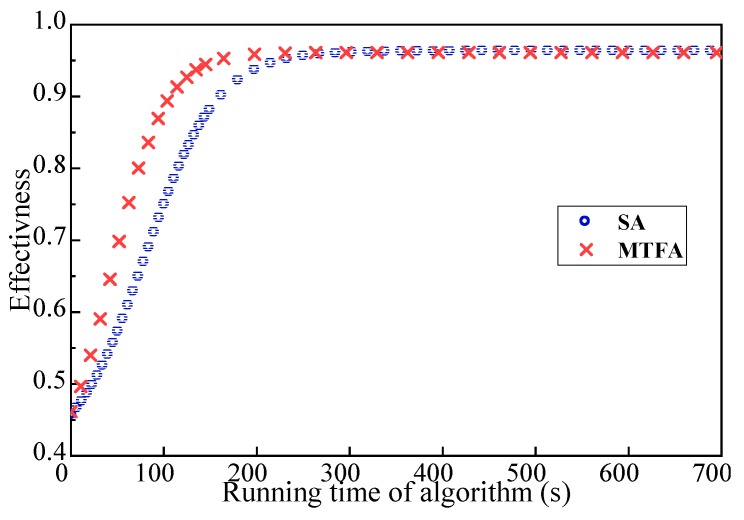
Effectiveness of teams found with the weighted synergy graph and MTFA; the size of the initial robot set is 15.

**Figure 15:**
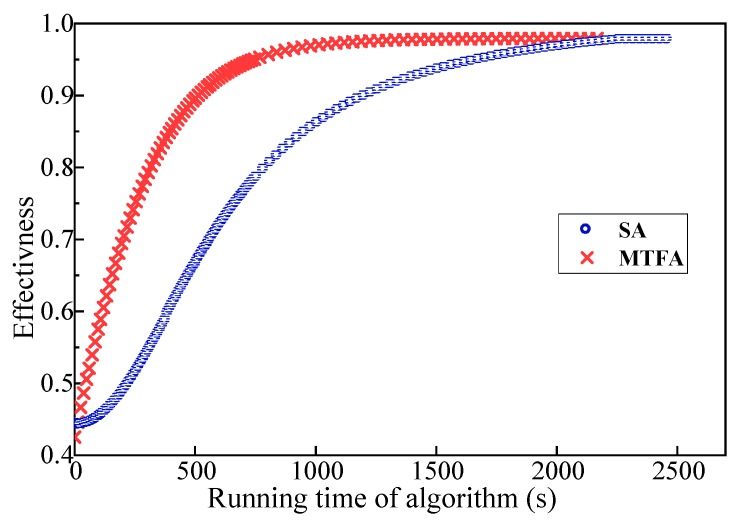
Effectiveness of teams found with the weighted synergy graph and MTFA; the size of the initial robot set is 35.

Figure 14 and Figure 15 show the relationship between the operation time and the effectiveness of the robot team. The size of the initial robot set is 15 and 35, respectively. In Figure 14 and Figure 15, the convergence rate of the MTFA’s optimal solution is much better than that of the original SA. Furthermore, the larger the size of the robot set, the more obvious the advantage of MTFA. In addition, the results of a larger robot set can be improved after removing the restriction of the computation time and the number of iterations.

### 5.2. Result of Field Experiments

These experiments were conducted at the test site of Wuhan University to verify the effectiveness of the MRCPS for sensing the environmental variables of a transmission line. This test site is used to simulate the working environment of the inspection robot.

**Figure 16:**
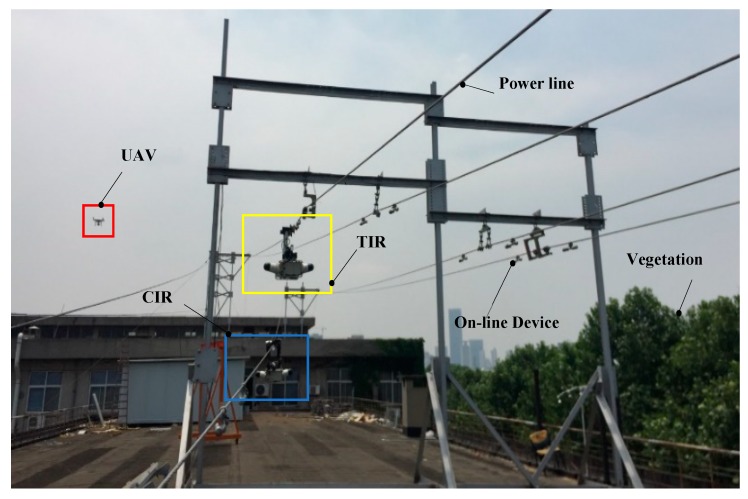
The field experiments, i.e., the Multi-Robot Cyber Physical System (MRCPS) composed of TIR, CIR and UAV performs an inspection task.

In order to simulate the open terrain around the overhead transmission line, a testing site with 50-m length and 30-m width is located on the top floor of the building. In addition, the analog line uses a more complex distribution than the actual power line. The transmission line of the test site integrates an adjustable number of on-line devices to cause spatial variations in environmental variables. Figure 16 shows an image of a field experiment.

In this work, both effects were fully validated: the effectiveness of collaborative work of the multi-robot in MRCPS, and the balance performance of the economy and collaboration capability of the multi-robot team in the multiple scenarios.

**Figure 17:**
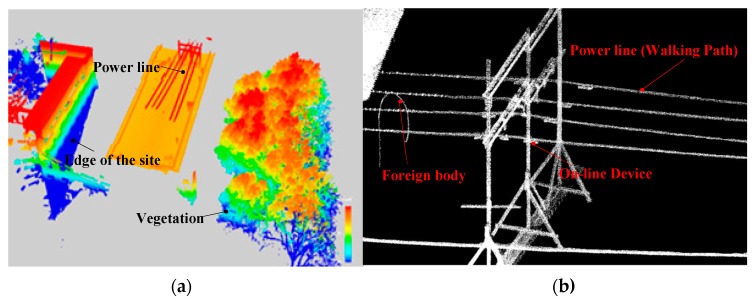
The obtained point cloud data and map of field testing site. (**a**) The point cloud data collected by the inspection robot; (**b**) The inspection map.

Firstly, the inspection robot carries the laser scanner and the Inertial Measurement Unit (IMU) equipment to measure the space parameters. The basic spatial data of the test site is obtained, such as the number of transmission lines, the length of each line, the type, number, and location of devices on the line, and the vegetation distance. With obtained spatial data, a multi-robot system inspection map and inspection database are then constructed. The obtained point cloud data and map are shown in Figure 17 (these data are cited from our previous articles [24,25,26], which have described the acquisition process and results in detail), in which Figure 17a is the point cloud data collected by the robot, and Figure 17b is the constructed inspection map.

Subsequently, scenario 1 was designed for verifying the effectiveness of the multi-robot team of the MRCPS. Table 5 shows the default experimental parameters of scenario 1. Table 6, Table 7, Table 8 and Table 9 shows the variation rules of the sub-scenarios 1.1–1.4. In the multiple scenarios, different objective task demands are proposed by changing the inspection distance, the number of inspection objectives, and the required inspection time.

**Figure 18:**
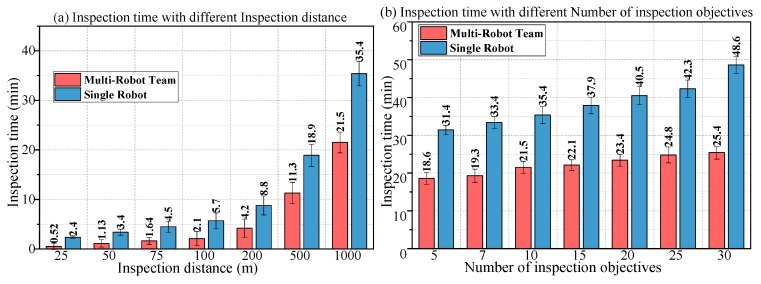

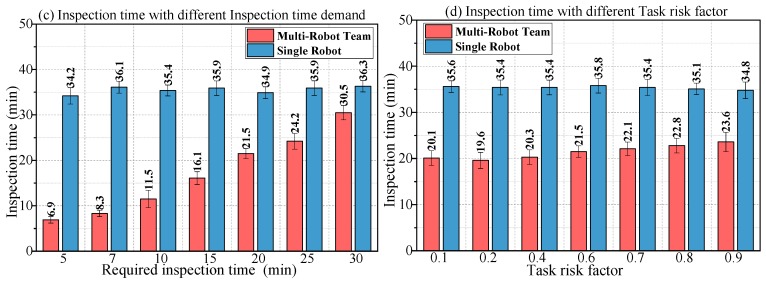
Results of scenario 1; inspection time with different parameters. (**a**) Inspection time with different inspection distance; (**b**) Inspection time with different number of inspection objectives; (**c**) Inspection time with different required inspection times; (**d**) Inspection time with different task risk factors.

Figure 18 shows the comparison of the inspection results of the multi-robot team with those of the individual robot in Scenario 1. In Figure 18a–d, the objective task demand and the task risk factor are adjusted to obtain the duration of the multi-robot team and the individual robot under different conditions.

The comparison between the robot team and the individual robot is shown in Figure 13. The following properties are summarized.

- Under the same conditions, the multi-robot team completed the inspection task in a much shorter time than the single robot.
- The inspection time of the multi-robot team was most affected by required inspection time *t_o_* and inspection distance *L*. The inspection time of the single robot was most affected by inspection distance *L* and the number of inspection objectives *n_o_*.
- The higher the required inspection time *t_o_*, the harder it was for the robot team to achieve the goal.

First of all, the collaborative inspection of MRCPS greatly reduces the time required for completing the task. Secondly, *t_o_* directly restricts the inspection time of the robot team and has the greatest impact on the results. In the test of the *L* change, the collaborative inspection rate is constant, and *t_o_* is changed indirectly, which makes the actual inspection time change obviously. However, the single robot is affected only by the difficulty of the inspection task (*L*, *n_o_*). Finally, due to the limitation of the initial size of the robot set, the team capability is constrained, i.e., the higher the requirement, the worse the performance will be.

Finally, the default experimental parameters for scenario 2 are shown in Table 10. The performance of cost and collaboration capability of the multi-robot team in MRCPS is adjusted by changing the objective task demand and the task risk factor. Table 11 and Table 12 shows the range of variation of the experimental parameters for sub-scenarios 2.1 and 2.2.

**Figure 19:**
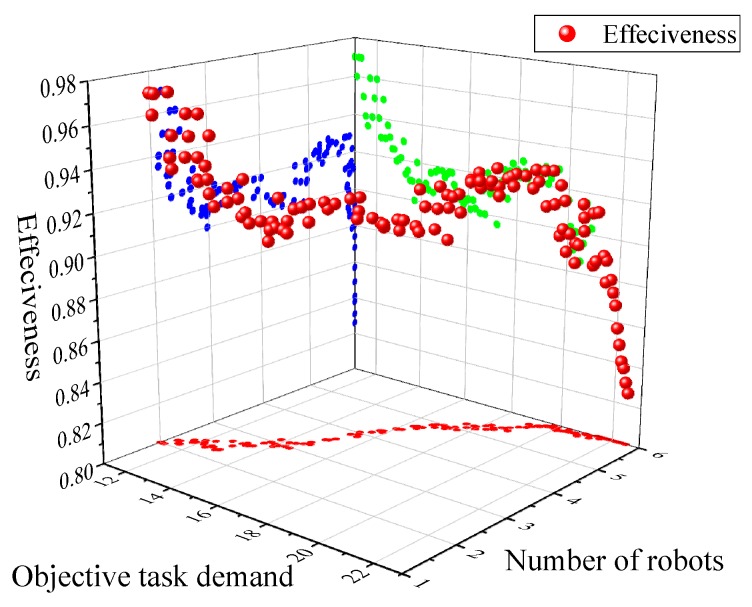
Results of scenario 2.1, the effectiveness of multi-robot team with different objective task demands.

**Figure 20:**
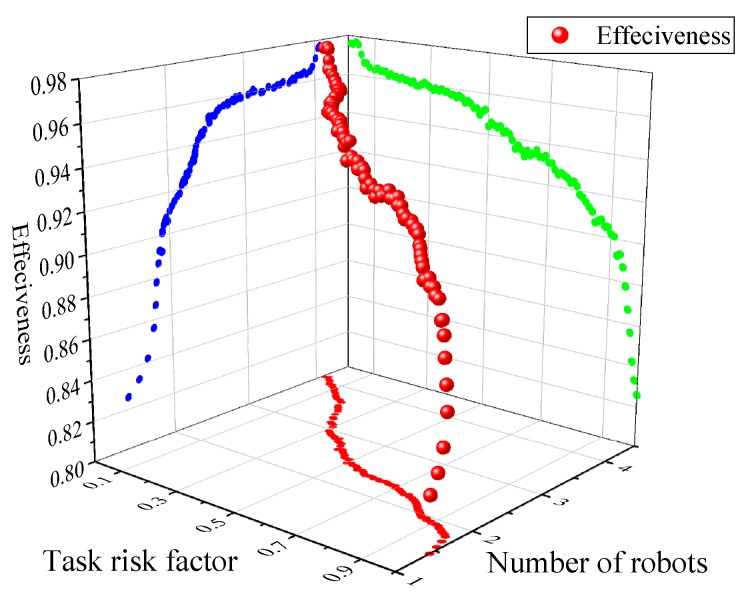
Results of scenario 2.2, the effectiveness of multi-robot team with different task risk factors.

Similar to the effectiveness of the team formation, the effectiveness of the multi-robot team in collaboration capability is calculated by the relationship between the actual inspection time and the required inspection time. The effectiveness of the multi-robot team in practical work is shown in Equation (19).

(19) $$Effectiveness=\frac{\left[t_{o}-|t_{o}-t_{Rx}|\right]}{t_{o}},$$

where *t_o_* is the required inspection time, and *t_Rx_* is the actual inspection time of the multi-robot team. Figure 19 shows the effectiveness of multi-robot team with different objective task demands, and the effectiveness of different task risk factors is shown in Figure 20.

It is seen from Figure 19 that the effectiveness of the robot team is negatively related to the objective task demand *μ_o_*; corresponding to the specified set of robots, especially the task demand is too low or too high, the variation of the working efficiency of the robot team is more obvious. For the moderate value of *μ_o_*, little change is observed in the effectiveness of the multi-robot team, and it can maintain a high efficiency (0.89–0.93). In addition, with the difficulty of the target task increasing, the number of robots is gradually increasing, that is, the economic efficiency declines the execution of the robot team.

The changing of the effectiveness of the MRCPS is more obvious under the influence of task risk factor *ρ* (as shown in Figure 20). The effectiveness of robot team is also negatively related to the task risk factors. There is no stable phase in the efficiency curve, and the change rate is large compared with the result in Figure 19. With the increase of task risk factor, the number of robots has steadily declined, and the economic efficiency has been gradually enhanced. That is, the higher the risk, the greater the return; the lower the risk, the greater the cost.

## 6. Discussion

The Multi-Robot Cyber Physical System (MRCPS) is derived from the rapid development and extensive application of robots, multi-agent theory, and the WSN. The most significant characteristic of the MRCPS is the dynamic monitoring of transmission lines by multiple mobile WSNs composed of heterogeneous robots and static nodes along a ground line. The monitoring mode of MRCPS is different from that of the traditional WSN with low flexibility and high cost of operation and maintenance; it is also different from single robot inspection system, whose fault tolerance is poor, and wireless signal could be frequently blocked by vegetation. WSN and multi-robot technologies have been rapidly developing in recent years, especially in terms of the light weight and cost-effectiveness of the hardware. Thus, it has become possible to build a multi-robot team and propose a new method for monitoring high-voltage transmission lines. The MRCPS, combining the characteristics of robots, WSN, and multi-agent theory, is a very distinctive system. Firstly, the system structure is changeable and flexible for mobile robots as the information sensing center; secondly, the system has the properties of high inspection quality and effectiveness; thirdly, as fewer nodes are deployed, the deployment cost is decreased, and the system’s fault-tolerant performance is improved. These characteristics of the MRCPS provide a strong technological support for the effective inspection of the transmission line.

The main contribution of the proposed method is to improve the intelligent level of the power grid monitoring system and the quality of the inspected data. At present, the single robot control and communication mode is commonly used for inspecting transmission lines. That is, there is only one robot per inspection task. Thus, such control and communication modes seriously restrict the large-scale application of the robotic line inspection. In this paper, a highly practical Multi-Robot Cyber Physical System (MRCPS) is proposed. The proposed method can greatly reduce the workload of operators and improve the intelligent level. Further, this research makes a new attempt in theory: the relationship model of the multi-robot is analyzed and summarized; the method of normal distribution is introduced to the statistics and calculation of robot task-based ability; the mathematical model of the multi-robot weighted graph is also introduced. Under the premise of guaranteeing working efficiency, by setting risk factors, the number of robots is reduced, and the economic effectiveness of tasks is improved. Finally, based on the mathematical model and simulated annealing algorithm, a Multi-robot Team Formation Algorithm (MTFA) is designed. It balances the relationship between the global optimal solution and the operation time, and also optimizes the working efficiency and operation cost. Therefore, the reliability and practicability of the MRCPS are greatly improved.

The proposed method improves the practical applications in the following aspects. Firstly, the simulation results verify that the multi-robot weighted graph model can effectively improve the efficiency of the robot team. Secondly, the Multi-robot Team Formation Algorithm (MTFA) can facilitate the convergence rate of the global optimal solution and improve the actual performance in application. Thirdly, through field experiments, it is found that the multi-robot team can effectively reduce the inspection time and improve the working efficiency. Finally, the multi-robot team can effectively balance the relationship between the inspection effectiveness and operation cost of the task by adjusting the task risk factors. To sum up, the proposed Multi-Robot Cyber Physical System (MRCPS) can efficiently monitor transmission lines and environmental variables.

## 7. Conclusions

This paper proposes a novel method of a Multi-Robot Cyber Physical System (MRCPS) for the inspection of transmission lines. The main conclusions are summarized as follows:

(1) The MRCPS is able to act as a new type of intelligent monitoring system to automatically inspect the transmission lines. Since the robot team can adapt to different scenarios by changing the organization structure and task assignment, it is more flexible and fault-tolerant than a single robot or WSN sensor system. In addition, the robot team can call on more resources to complete the task, which makes the system more efficient. When a robot team is replaced with a large number of static nodes, the availability and the cost of the monitoring system can be improved.
(2) The proposed method mainly includes two parts, i.e., the multi-robot weighted graph model and the MTFA. In the first part, the composition and structure of the robot team are introduced. Then, the characteristics of the relationship between robots are analyzed, and the weighted graph model is proposed. Finally, a new mathematical model for the robot team is proposed. The proposed model fully considers the characteristics of the relationship and ability of robots, and a scheme is designed to balance the working efficiency and operation cost. In the next part, a new algorithm (MTFA) is designed. The MTFA is similar to the Simulated Annealing algorithm, as it is designed to find the global optimal solution of the objective function. Then, the mathematical model is combined to update the weight of the links and conversion to objective function. Finally, the robot team risk factors are ranked.
(3) In simulations and experiments, multiple experimental scenarios are performed for verifying the two kinds of effectiveness. It not only verifies the effectiveness of the weighted synergy graph model and the MTFA, but also the effectiveness of the multi-robot team in different scenarios. The experimental results show that the multi-robot weighted synergy graph and MTFA can find the optimal robot team, and they perform better than other methods. In the comparison experiment, the inspection efficiency of robot team for transmission line is much better than that of a single robot. Furthermore, the multi-robot team can effectively balance the performance of work effectiveness and operation cost.

Further research should mainly focus on two aspects. One is that the weighted graph model has not designed the variability of the transitivity of the relations among robots. As the relations (communication efficiency) change, the optimal result is less reliable. In addition, more factors are need to be calculated to regulate the relation link between robots. The other is that the MTFA sometimes cannot achieve ideal result rapidly; it is more difficult to find the optimal robot team if the initial robot set is huge. Multivariate analysis is required to control the convergence of the global optimal solution.

## Figures and Tables

**Table 1:** Characteristic parameters obtained by different detection methods. LiDAR: Light Detection and Ranging.

Parameter Class	Inspection Target	Phenomenon	Sensing Methods
Shape	Line	Untwisted or broken strand	Visible light imaging sensing
		Foreign body coverage	Visible light imaging sensing
	On-line device	Color variation	Visible light imaging sensing
		Aging, corrosion	Visible light imaging sensing
Environment	Line	Covering foreign bodies	Visible light imaging sensing
		Galloping	Visible light imaging sensing
	On-line device	Color variation	Visible light imaging sensing
	vegetation	Distance change	visible light imaging/LiDAR
	climate	Meteorological change	Other sensing
Energy	Line	Value anomaly	infrared imaging sensing
	On-line device	Value anomaly	infrared imaging sensing
Space	Line	Galloping	visible light imaging sensing
		Sag change	visible light imaging/LiDAR
	On-line device	Displacement	visible light imaging/LiDAR
	vegetation	Distance change	visible light imaging/LiDAR

**Table 2:** Robots for transmission lines.

References	Institutions	Application	Scenario
[3,9,10,23]	Hydro-Québec (Canada)	Monitoring, Maintenance (Deicing )	Overhead transmission line
[14,15]	HiBot (Japan)	Monitoring	Overhead transmission line, Power tunnel
[11,12,13,20,24,25,26,27]	Wuhan university (China)	Monitoring, Maintenance (Deicing, Replacement)	Overhead transmission line
[19,21]	Chinese Academy of Sciences (CAS, China)	Monitoring	Overhead transmission line
[6,16]	Shanghai university (China)	Monitoring	Overhead transmission line
[4]	Electric Power Research Institute (EPRI USA)	Monitoring	Overhead transmission line
[17,22]	Eletrobras-Cepel (Brazil)	Monitoring	Overhead transmission line
[5,8]	University of KwaZulu-Natal (South Africa)	Monitoring	Overhead transmission line

**Table 3:** Detection sequences of waiting-inspection targets.

Detection Sequence	Line Number	Inspection Point	Coordinate	Class
①	A-phase	A	X_A_, Z_A_	Shape
②	Ground wire	B	X_B_, Z_B_	Shape
③	C-phase	C	X_C_, Z_C_	Environment
④	B-phase	D	X_D_, Z_D_	Space
⑤	A-phase	E	X_E_, Z_E_	Shape
⑥	C-phase	E	X_E_, Z_F_	Environment

**Table 4:** Key parameters of inspection robots and unmanned aerial vehicles (UAV). PTZ: pan–tilt–zoom.

Robot	PIR	TIR	CIR	UAV (DJ-Innovations, DJI Phantom 4)
**Specifications**				
Dimensions (mm)	510 × 270 × 156	910 × 420 × 816	950 × 420 × 1100	289.5 × 289.5 × 196
Weight	15.2 kg	44.8 kg	56.6 kg	1.38 kg
Speed	0.65 m/s	0.72 m/s	0.52 m/s	20 m/s
Autonomy	3 h	8.5 h	7.8 h	28 min
Charge	2.5 h	6 h	6 h	1 h
Load capacity	5 kg	20 kg	25 kg	/
**Equipment**				
Cameras	PTZ camera	PTZ × 2 cameras	PTZ × 2 cameras	Down camera
Sensors	Temperature, humidity, inclination	Temperature, humidity, inclination	Temperature, humidity, inclination	/
other	Infrared camera	Infrared camera, Laser radar, Inertial Measurement Unit/Global Positioning System (IMU/GPS)	Infrared camera, Laser radar, IMU/GPS	/

**Table 5:** Default parameters of field experiment for scenario 1.

Parameter	Default Value
Test area size (m × m)	50 × 30
Required inspection time *t_o_* (s)	1200
Inspection distance *L* (m)	1000
Number of inspection objectives *n_o_*	10
Objective task demand *μ_o_*	8.3
Task risk factor *ρ*	0.6
Size of robots set *N*	6

**Table 6:** Parameters of Scenario 1.1.

Parameter	Setting 1	Setting 2	Setting 3	Setting 4	Setting 5	Setting 6	Setting 7
*L* (m)	25	50	75	100	200	500	1000
*t_o_* (s)	0.02 × *L*	0.02 × *L*	0.02 × *L*	0.02 × *L*	0.02 × *L*	0.02 × *L*	0.02 × *L*

**Table 7:** Parameters of Scenario 1.2.

Parameter	Setting 1	Setting 2	Setting 3	Setting 4	Setting 5	Setting 6	Setting 7
*n_o_*	5	7	10	15	20	25	30

**Table 8:** Parameters of Scenario 1.3.

Parameter	Setting 1	Setting 2	Setting 3	Setting 4	Setting 5	Setting 6	Setting 7
*t_o_* (min)	30	25	20	15	10	7	5

**Table 9:** Parameters of Scenario 1.4.

Parameter	Setting 1	Setting 2	Setting 3	Setting 4	Setting 5	Setting 6	Setting 7
*ρ*	0.1	0.2	0.4	0.6	0.7	0.8	0.9

**Table 10:** Default parameters of field experiment for scenario 2.

Parameter	Default Value
Test area size (m × m)	50 × 30
Required inspection time *t_o_* (s)	1200
Inspection distance *L* (m)	1000
Number of inspection objectives *n_o_*	15
Objective task demand *μ_o_*	12.5
Task risk factor *ρ*	0.6
Size of robots set *N*	6

**Table 11:** Parameters of scenario 2.1.

Parameter	Parameter Variation Range	Parameter Variation Amplitude
*μ_o_*	10–22.5	0.1

**Table 12:** Parameters of Scenario 2.2.

Parameter	Parameter Variation Range	Parameter Variation Amplitude
*ρ*	0.01–0.99	0.01
